# Effect of Transient Inactivation of Ventral Tegmental Area on the Expression and Acquisition of Nicotine-Induced Conditioned Place Preference in Rats

**DOI:** 10.6091/ibj.1402.2015

**Published:** 2015-10

**Authors:** Durna Chalabi-Yani, Hedayat Sahraei, Gholam Hossein Meftahi, Seydeh Bentolhuda Hosseini, Sara Sadeghi-Gharajehdaghi, Hengameh Ali Beig, Zahra Bourbour, Mina Ranjabaran

**Affiliations:** 1 *Dept. of Biology, School of Biological Sciences, Islamic Azad University, North Tehran Branch, Tehran, Iran; *; 2 *Neuroscience Research Center, Baqiyatallah (a.s.) University of Medical Sciences, Tehran, Iran*

## Abstract

**Background:**

Nicotine can activate dopaminergic neurons within the ventral tegmental area (VTA). However, there is no evidence about complete inhibition of VTA on nicotine reinforcement.

**Methods:**

in the present study, we used conditioned-place preference (CPP) method to study the effect of transient inhibition of left and/or right side of the VTA by lidocaine on nicotine reward properties. Male Wistar rats seven days after recovery from surgery and cannulation were conditioned to nicotine (1.5 mg/kg) in an unbiased designed CPP apparatus. Five min before each nicotine injection in conditioning phase, lidocaine (2%) was administered either uni- or bi-laterally into the VTA (0.5µl/rat).

**Results:**

results revealed that lidocaine administration into the left but not right side of the VTA reduced nicotine CPP significantly. The reduction was potentiated when lidocaine injected in to both sides of the VTA. In addition, the number of compartment crossing was reduced when lidocaine injected in both side of VTA as well as left side. On the other hand, rearing was reduced when lidocaine injected to the right but not left side of VTA. At last, sniffing was reduced only in the group in which received lidocaine in both side of VTA. Sniffing and rearing increased in the group in which received lidocaine in right side.

**Conclusion:**

It is concluded that the right and left side of VTA play different role in nicotine-induced activity and reward.

## INTRODUCTION

Despite effective public health campaigns that have decreased tobacco use, smoking remains a predominant health troublesome. Smoking is essentially a myriad of behaviors reflecting addiction to nicotine, the major neuroactive ingredient of tobacco [1]. Several studies have shown that nicotine activates acetylcholine nicotinic receptors to initiate a series of adaptive changes at the cellular and circuit levels in brain, particularly in the ventral tegmental area (VTA) [2-4]. One important pathway affected by nicotine and other drugs of abuse is dopaminergic originating in VTA [5-7]. VTA dopaminergic neurons contribute to the rewarding and addictive properties of psycho-stimulants, including nicotine [3]. In the VTA, nicotine increases the activity of dopaminergic neurons and dopamine release in the nucleus accumbens as well as the prefrontal cortex, a phenomenon widely correlated with drug reward or reinforcement [5]. Various nicotinic receptor subtypes are widely expressed in the VTA in both dopaminergic projection neurons and local GABA-ergic interneurons [8, 9]. Nicotine can increase dopamine release in the nucleus accumbens by means of its α2β4 receptor [3]. However, it has been shown that the VTA contains another cell types other than dopaminergic and GABA-ergic neurons, including peptidergic neurons and neurons-containing nitric oxide [10]. However, the potential contribution of nicotine to VTA dopaminergic neurons is unknown. Insights into nicotine-mediated activation of this important brain region will significantly help to understand the underlying mechanisms of nicotine addiction. Moreover, to our knowledge, there is no study dealing with the effects of complete transient inactivation of the VTA on nicotine reward. Therefore, the goal of this study was to determine whether transient inactivation of the VTA inhibits nicotine rewards. In addition, several dopamine-related behaviors, including sniffing, rearing, and locomotion were considered. 

## MATERIALS AND METHODS

***Animals*** ***. ***Male Wistar rats (300 ± 50 g, Pasture Institute of Iran, Tehran) were used in all experiments (6-8 rats for each experiment). Animals were kept on a standard 12/12 h lightcycle with *ad libitum* food and water. All procedures were conducted in accordance with standard ethical guidelines and approved by the local ethics ccommittee (The Baqiyatallah University of Medical Committee on the Use and Care of Animals, 81/021, July 10, 2002). 

***Apparatus*** ***. ***Two-compartment conditioned-place preference (CPP) apparatus (30 × 60 × 30 cm) was used in these experiments. Place conditioning was conducted using an unbiased procedure, with minor changes to the design previously described [11-13]. Briefly, the apparatus was made of wood and both compartments were identical in size (the apparatus was divided into two equal-sized compartments by means of a removable white guillotine door) and shading (both were white), but distinguishable by texture and olfactory cues. For preparation of the tactile difference between the compartments, one of the compartments had a smooth floor, while the other one had a nylon white mesh floor. A drop of menthol was placed at the right center of the compartment with a textured (nylon mesh) floor to provide the olfactory difference between the compartments. Two compartments were differently striped black on their sides. In this apparatus, rats showed no consistent preference for either compartment, which supports our unbiased CPP paradigm.

***Surgical procedures*** ***and lidocaine*** ***injection.*** The surgical area was shaved. Animals were placed in a stereotaxic frame (Stoelting, Wood Dale, IL, USA), and then a small incision was made in the scalp to expose the skull. Using bregma and lambda as landmarks, the skull was leveled in the coronal and sagittal planes. Holes were drilled in the skull at the anteroposterior (in reference to bregma) and the mediolateral coordinates that correspond to the VTA  (-3.3 mm anteroposterior, ± 0.5 mm mediolateral, and 8 mm from top of the skull) based on the Paxinos and Watson [14]. One or two stainless steel cannulas (23 gauges) was/were placed stereotaxically (Stolting instruments, USA) into the VTA. Lidocaine (dissolved in sterile saline) was delivered via a 30-gauge blunt tapered needle (0.25 µl/side) at a rate of 0.5 µl/min. The injection needle was left in place for 5 min after injection and then removed.

***Drugs.*** The following drugs were used: nicotine hydrogen tartrate (Tocris, UK), lidocaine and diazepam hydrochloride (Sigma, CA, USA), and ketamine hydrochloride (Alfasan, Netherlands). All drugs were dissolved in physiologic saline (0.9%) just before the experiments. Control groups received physiologic saline. 

***Behavioral testing*** ***.*** CPP consisted of three phases: pre-conditioning, conditioning, and post-conditioning.

***Pre-conditioning.*** On the first day (pre-exposure), each rat was placed separately into the apparatus for 10 min with free access to all compartments. 

***Conditioning.*** This phase consisted of a three-day schedule of conditioning sessions. In this phase, animals received three trials in which they experienced the effects of the drugs while confined in one compartment for 45 min as well as three trials in which they experienced the effects of saline while confined in the other compartment. Access to the compartments was blocked on these days.

***Post-conditioning phase.*** On the fifth day (the preference test day), the partition was removed, and the rats could access the entire apparatus. The mean time that each rat spent in either compartment during a 10 min period was determined as the preference criterion. No injection was given during the acquisition tests.

***Locomotors activity.*** Locomotors activity was measured in two main compartments during the testing phase. For this purpose the ground area of the compartments were divided into four equal-sized squares. Locomotion was measured as the number of crossings from one square to another during 10 min. The doses of drugs used in these experiments did not alter locomotion activity. In addition, the number of compartment crossing during test phase was also recorded as another index of animals’ activity, which was not statistically significant between the groups (data not shown). 

**Fig. 1:**
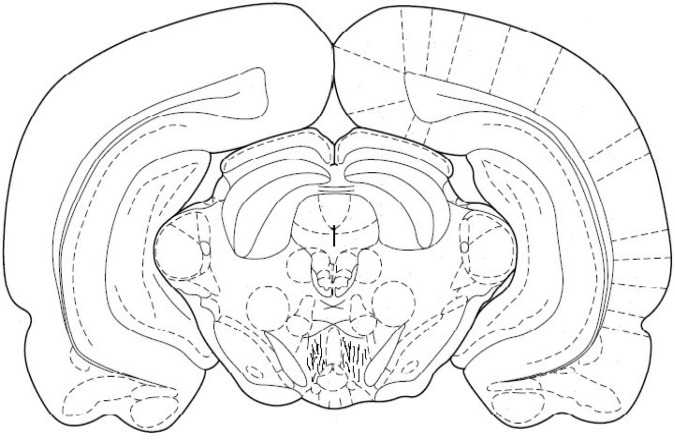
Location of cannula tips in the VTA. The lines indicate where the cannula tips were placed

***Histology.*** After the completion of testing, all animals were anesthetized and received a transcardiac perfusion with 0.9% normal saline, followed by 10% buffered formalin. The brains were removed, blocked and cut coronally in 40 µm sections through the cannula placements. The tissues were stained with cresyl violet and examined by light microscopy by an observer unfamiliar with the behavioral data. Only the animals with correct cannula placements were included in the data analysis (Fig. 1).

***Statistic.*** Data were expressed as mean ± S.E.M. Nicotine dose-response was analyzed using one-way analysis of variance (ANOVA), followed by Tukey's post hoc. A three-way ANOVA was applied for the analysis the differences between the lidocaine-treated groups, pretreatment and treatment as factors. When three-way ANOVA showed a significant difference, the Tukey HSD test was applied to demonstrate the difference. Differences with *P* < 0.05 were considered significant.

## RESULTS

***Effect of nicotine on place preference:***

**Fig. 2:**
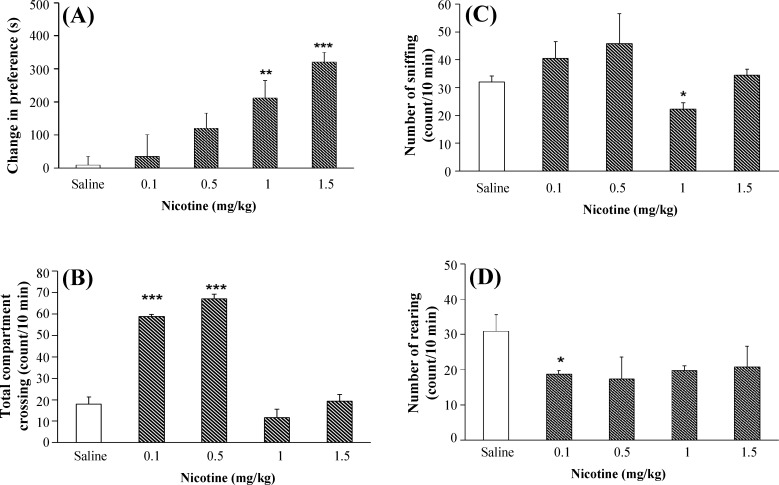
Effect of different doses of nicotine on place preference and dopamine-related behaviors in rats. Animals receivd nicotine (0.1, 0.5, 1, and 1.5 mg/kg, i.p.). Each point represents the mean ± SEM of conditioning score for 6-8 rats,^ *^P < 0.05 , ^**^P < 0.01, and ^***^P < 0.001 are different from the saline control group

***Nicotine-induced place preference dose-response. ***The effect of nicotine on place preference is shown in Figure 2A. Application of nicotine (1 and 1.5 mg/kg) produced significant place preference to the drug-paired compartment (F [5, 30] = 3.21, *P* < 0.01). Nicotine at doses of 0.1 and 0.5 mg/kg significantly increased locomotor activity (F [4, 25] = 4.89, *P* < 0.01) (Fig. 2B). The nicotine also reduced sniffing at a dose of 1 mg/kg (F [4, 25] = 2.39, *P* < 0.05) compared with the control (saline) group (Fig. 2C). In addition, nicotine significantly reduced rearing at a dose of 0.1 mg/kg (Fig. 2D). 

**Fig. 3:**
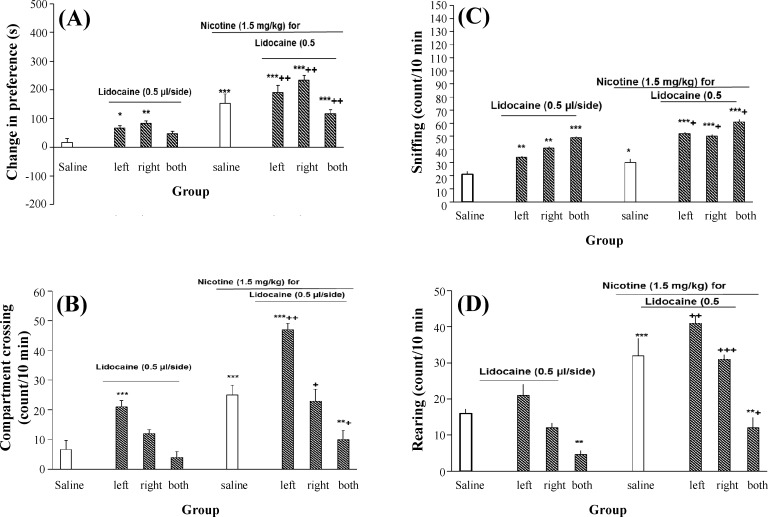
Effect of left, right, and/or both side transient inhibition of VTA on nicotine place preference. Animals received lidocaine into their VTA before nicotine (1.5 mg/kg, i.p.), or saline (1 ml/kg) in each conditioning sessions. (A) Injection of lidocaine into the right, left, or both sides of the VTA cannot inhibit nicotine-induced place preference. Each point shows the mean ± SEM of conditioning score for 7-8 rats. ^*^P < 0.05, ^**^P < 0.01, and ^***^P < 0.01 indicate differences with the saline control group, and ^++^*P *< 0.001 shows differences with the lidocaine control group. (B-D) The inhibitory effect of transient inactivation of the left, right, or both sides of VTA on dopamine-related behaviors in rats. Transient inhibition of VTA resulted in a significant increment of locomotor activity as shown by compartment crossing (B). In addition, the number of sniffing (C) and rearing (D) also was increased in the animals. Each point shows the mean ± SEM of the behavior for 7-8 rats. ^*^P < 0.05, ^**^P < 0.01, and ^***^P < 0.01 are different from the saline control group and ^+^*P* < 0.05, ^++^*P* < 0.01, and ^+++^*P* < 0.001 are different from lidocaine control group. VTA, ventral tegmental area

***Application of lidocaine in VTA:***

***Nicotine place conditioning paradigm in rats when right or left and/or both sides VTA was transiently inactivated. ***The results showed that application of lidocaine in the right or left side or both parts of VTA did not significantly change nicotine-induced place preference (three-way ANOVA within-group comparison: side effect: F [5, 35] = 0.16, *P* > 0.05, pretreatment effect: F(1, 35) = 0.98, *P* > 0.05, treatment effect: F [5, 35] =2.61, *P* < 0.01, side × pretreatment × treatment effect: F [8, 73] = 5.32, *P* < 0.001]) (Fig. 3A). Moreover, inhibition of the left side of VTA significantly increased total compartment crossing in the nicotine-treatment rats, whereas inhibition of the right part of VTA or both sides significantly reduced the animals locomotion (three-way ANOVA within-group comparison: side effect: F [5, 35] = 3.67, *P* < 0.01, pretreatment effect: F [1, 35] = 4.21, *P* < 0.01, treatment effect: F [5, 35] = 4.12, *P* < 0.01, side × pretreatment × treatment effect: F [8, 73] = 5.45, *P* < 0.001]) (Fig. 3B). In relation to sniffing, the behavior increased when left, right or both sides of VTA inhibited (three-way ANOVA within-group comparison: side effect: F [5, 35] = 3.67, *P* < 0.001, pretreatment effect: F [1, 35] = 4.35, *P* < 0.01, treatment effect: F [5, 35] = 5.43, *P* < 0.01, side × pretreatment × treatment effect: F [8, 73] = 5.9, *P* < 0.01) (Fig. 3C). Inhibition of the left or right side of VTA increased the number of animals rearing in the nicotine-treated rats. However, the number of animals rearing significantly decreased when both sides of VTA inhibited (three-way ANOVA within-group comparison: side effect: F [5, 35] = 1.09, *P* > 0.05, pretreatment effect: F [1, 35] = 0.79, *P* > 0.05, treatment effect: F [5, 35] = 1.21, *P* < 0.05, side × pretreatment × treatment effect: F[8, 73] = 3.21, *P* < 0.01) (Fig. 3D). 

## DISCUSSION

The major finding of the present study is that the both sides of the VTA have different contribution on nicotine-induced CPP, which is corroborated by means of the changes in dopamine-dependent behaviors such as locomotion, rearing, and sniffing. To our knowledge, previous studies did not consider the effects of complete transient inactivation of the VTA on nicotine-induced rewarding properties. However, the present study, for the first time, attempts to evaluate these behavioral changes alongside the nicotine-induced CPP. 

Our results showed that lidocaine administration to the left and/or right sides of VTA increased the time spend in the drug-paired compartment. In addition, administration of lidocaine to the right side of the VTA was more effective for place preference enhancement than the left side. It is likely that the right side of the VTA has an effective inhibitory role in the rewarding properties of nicotine, and the two sides of the VTA probably have different roles in euphoric effects of nicotine. 

Furthermore, our results showed that administering lidocaine simultaneously to the both sides of the VTA could not completely inhibit the CPP induced by nicotine. This result may be explained that nicotine acts through two pathways: first, activation of dopaminergic neurons of VTA and second, activation of the dopaminergic terminals in the nucleus accumbens [3]. Lidocaine may inhibit the cell bodies of dopaminergic neurons in the VTA, and nicotine induced CPP by means of dopaminergic terminals in the nucleus accumbens. 

Our data revealed that dopamine-related behaviors were increased in low doses of nicotine, whereas decreased in high doses. In other words, nicotine may produce changes in the brain and then suppress dopamine-dependent behaviors in the animals. This inhibitory effect of nicotine was dose dependent, suggesting that different doses of nicotine may exert different influences on the mesolimbic system after drug abuse. Therefore, these behaviors may be useful devices as an auxiliary criterion for studying the rewarding effects of nicotine and possibly other addictive drugs [15-18]. In CNS, nicotine activates neuronal nicotinic acetylcholine receptors, particularly in the dopaminergic neurons within the VTA [3]. It has been shown that some subtypes of these receptors such as α_2_β_4_ mediate the influx of Na^+^ ions. When these receptors were activated by nicotine, influx of Na^+^ enhances the membrane potential and leads to greater dopamine release from vesicles in the targets of the VTA such as nucleus accumbens [19]. Moreover, nicotine activated the dopamine pathways in hippo-campus and amygdale and enhanced memory [20-22]. 

Moreover, our data showed that administration of lidocaine to the left side of VTA increased locomotion in the animals. However, the increased locomotion with administering lidocaine to the right side was not statistically significant. Increased locomotion by inhibition of left or right side of VTA indicated over activity of the dopamine mesolimbic system [3, 4, 23]. Administering lidocaine simultaneously to both sides of VTA led to decreased locomotion, which was not significant as compared to the control group. Furthermore, administration of lidocaine to the left side of VTA conditioned with nicotine led to an intensive increase in animal’s locomotion, whereas administer-ing lidocaine to both sides of animals received nicotine resulted in considerable decrease in locomotion. These findings indicated that the right side of VTA probably has the major role in dopamine-dependent activities; this role is reinforced with administration of nicotine. In addition, inhibition of both sides of the VTA leads to decreased locomotion in the animals which is almost consistent with the decrease in CPP. Similarly, the number of rearing increased in animals with inhibited left VTA, while it decreased in animals with inhibition of both sides of the VTA. These changes in rearing are relatively in line with changes in locomotion and CPP which indicates that the inhibition of VTA may reinforce the dopamine-dependent activities in the drug-free state. Administering lidocaine to those animals received nicotine yielded similar results with greater intensity compared to the control group, showing the role of nicotine in intensive activation of the pathway related to the VTA. In conclusion, nicotine exerts its effect (at least partially and not completely) through activation of VTA. Moreover, left and right sides of the VTA have different contribution to dopamine-dependent behaviors in animals received nicotine. Furthermore, it seems that the inhibition of VTA may lead to the CPP or increased sniffing, locomotion, and rearing.
